# CT patterns and serial CT Changes in lung Cancer patients post stereotactic body radiotherapy (SBRT)

**DOI:** 10.1186/s40644-022-00491-1

**Published:** 2022-09-16

**Authors:** Rashid Al-Umairi, Usman Tarique, Rahim Moineddin, Laura Jimenez-Juan, Lan Chau Kha, Patrick Cheung, Anastasia Oikonomou

**Affiliations:** 1 Department of Medical Imaging, Sunnybrook Health Sciences Centre, University of Toronto, 2075 Bayview Avenue, Toronto, ON M4N3M5 Canada; 2 Department of Family and Community Medicine, Dalla Lana School of Public Health, University of Toronto, 500 University Avenue, Toronto, ON M5G1V7 Canada; 3 Department of Medical Imaging, St. Michael’s Hospital, University of Toronto, 30 Bond St, Toronto, ON M5B 1W8 Canada; 4 Department of Radiation Oncology, Sunnybrook Health Sciences Centre, University of Toronto, 2075 Bayview Avenue, Toronto, ON M4N3M5 Canada

**Keywords:** Computed tomography, Lung cancer, Stereotactic body radiotherapy, Radiation induced lung injury, CT patterns

## Abstract

**Background:**

To evaluate computed tomography (CT) patterns of post-SBRT lung injury in lung cancer and identify time points of serial CT changes.

**Materials and methods:**

One hundred eighty-three tumors in 170 patients were evaluated on sequential CTs within 29 months (median). Frequencies of post-SBRT CT patterns and time points of initiation and duration were assessed. Duration of increase of primary lesion or surrounding injury without evidence of local recurrence and time to stabilization or local recurrence were evaluated.

**Results:**

Post-SBRT CT patterns could overlap in the same patient and were nodule-like pattern (69%), consolidation with ground glass opacity (GGO) (41%), modified conventional pattern (39%), peribronchial/patchy consolidation (42%), patchy GGO (24%), diffuse consolidation (16%), “orbit sign” (21%), mass-like pattern (19%), scar-like pattern (15%) and diffuse GGO (3%). Patchy GGO started at 4 months post-SBRT. Peribronchial/patchy consolidation and consolidation with GGO started at 4 and 5 months respectively. Diffuse consolidation, diffuse GGO and orbit sign started at 5, 6 and 8 months respectively. Mass-like, modified conventional and scar-like pattern started at 8, 12 and 12 months respectively. Primary lesion (*n* = 11) or surrounding injury (*n* = 85) increased up to 13 months. Primary lesion (*n* = 119) or surrounding injury (*n* = 115) started to decrease at 4 and 9 months respectively. Time to stabilization was 20 months. The most common CT pattern at stabilization was modified conventional pattern (49%), scar-like pattern (23%) and mass-like pattern (12%). Local recurrence (*n* = 15) occurred at a median time of 18 months.

**Conclusion:**

Different CT patterns of lung injury post-SBRT appear in predictable time points and have variable but predictable duration. Familiarity with these patterns and timeframes of appearance helps differentiate them from local recurrence.

## Introduction

Lung cancer is the leading cause of cancer-related death worldwide accounting for approximately 13% of newly diagnosed cancers [1]. Surgery remains the treatment of choice for early-stage non-small cell lung cancer (NSCLC) with overall 5-year survival rate of approximately 50–70% [2]. Surgical intervention is feasible in patients who can tolerate the procedure and have a tumor where complete resection is plausible. However, impaired pulmonary function and advanced age limit patients’ eligibility for surgical intervention [3, 4]. The median survival rate amongst untreated patients with T1 and T2 lung cancer are 13 and 8 months respectively with 5-year cancer specific survival rate being 16% [5]. Therefore, an alternative therapeutic option for inoperable lung cancer is warranted. Traditionally, radiation therapy, including conventional and stereotactic body radiation therapy (SBRT), has been the alternative therapeutic option. Conventional radiotherapy uses different techniques to deliver fractionated radiation doses of 1.8–2.0 Gy/day over a treatment course of 8 weeks. However, conventional radiotherapy results are inferior to those of surgery [5]. SBRT has emerged as a new radiotherapy modality for the treatment of lung cancer. It involves a combination of stereotactic localization techniques and high dose hypofractionation which allows a delivery of high radiation dose to a target volume, while minimizing the radiation dose to adjacent lung. Typically, 3–5 fractions of 10–20 Gy are delivered over a period of 1–2 weeks [6]. Over the past decade, SBRT has become the modality of choice for treatment of inoperable early-stage non-small cell lung carcinoma (NSCLC) and has improved the population-based survival in stage I NSCLC [7, 8]. In 2010, Radiation Therapy Oncology Group (RTOG) published the results of a phase II prospective study (0236) of early NSCLC treated with SBRT, which showed perfect local tumor control of 97.8% and an overall survival of 55.8% at 3 years [9]. This, along with many other publications has led to widespread use of SBRT as a modality of treatment for inoperable early-stage NSCLC [10].

However, SBRT induces lung injuries which render the interpretation of follow-up chest computed tomography (CT) difficult. Although, some studies have reported post-radiation pattern of lung injury on follow-up CT [11–13], it remains challenging to distinguish post radiation changes from tumor recurrence. To the best of our knowledge, there is limited information in the literature about the timeline and duration of the different CT patterns of lung injury post-SBRT. Furthermore the changes in size of the treated lesion as opposed to the changes of the combined treated lesion and adjacent inseparable radiation changes has not been previously described separately. The purpose of this study is to evaluate the different CT patterns of post-SBRT changes in lung cancer patients and to define an expected timeframe of serial CT changes.

## Material and methods

The Research Ethics Board of our institution approved this retrospective single institution study.

### Patients

**Fig. 1:**
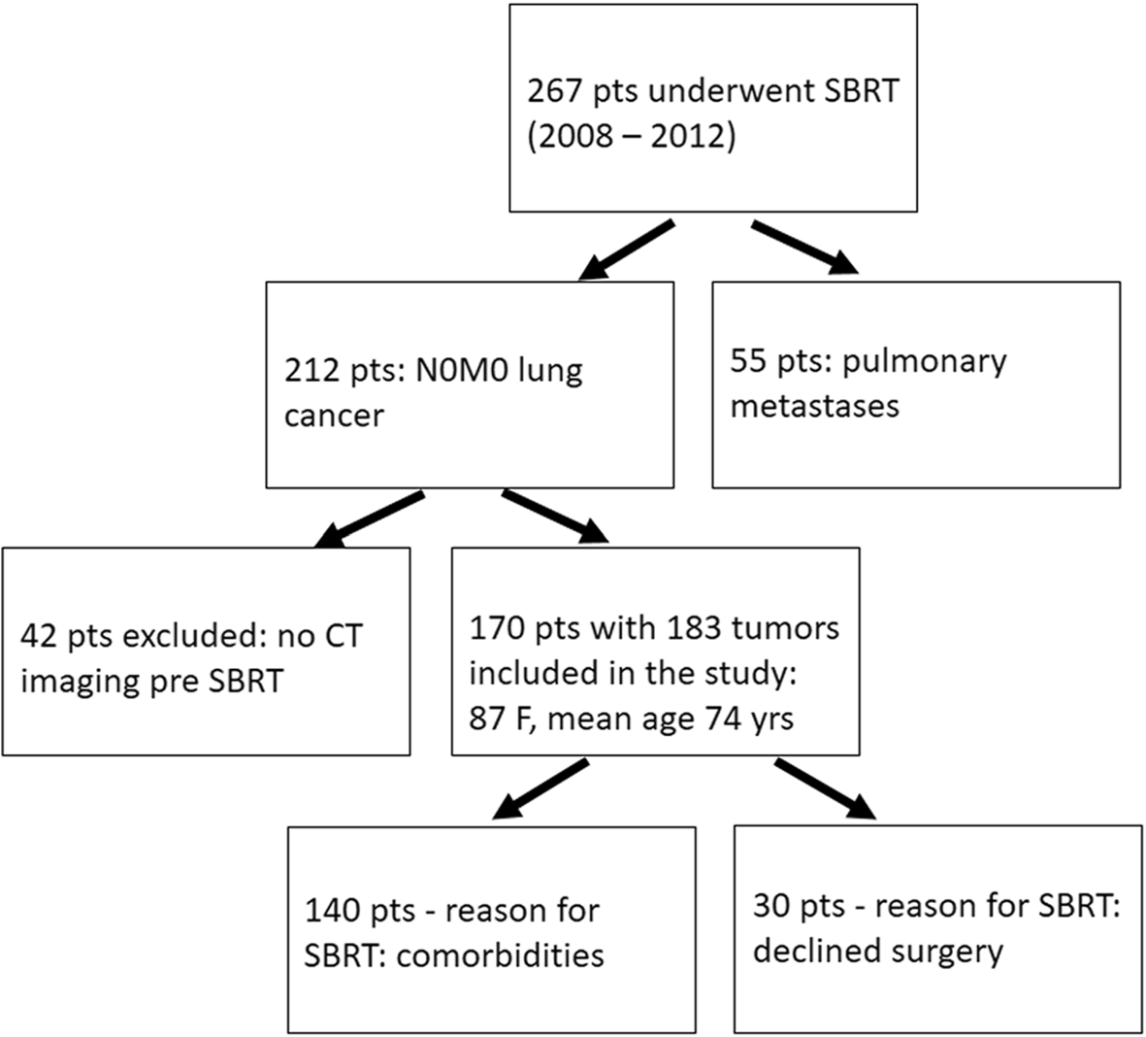
The flow chart describes the number of patients that underwent SBRT in our institution between 2008 and 2012 and the inclusion and exclusion criteria of the study

This retrospective study was based on a cohort of patients used for another research project that investigated the prediction of survival outcomes of SBRT treated lung cancer patients based on radiomics analysis [14]. This study included lung cancer patients treated with SBRT in a single institution (Fig. 1). All the patients included in the study had baseline staging PET/CT imaging before the SBRT treatment. Clinical data about tumor histology, primary tumor size, prescribed biological effective dose, age, gender, initial stage, were obtained from the institutional database and are summarized in Table 1.

**Table 1:** Characteristics of the patients, lesions and treatment

Patients	170
Male/Female	83/87
Age	74 (46–92)
T-stage	
T1aN0	69
T1bN0	58
T2aN0	33
T2bN0	6
T3 N0	11
T4 N0	6
Histology	143
Adenocarcinoma	78
Squamous	40
Large cell	2
NSCLC undifferentiated	11
non-diagnostic biopsy	12
Not biopsy proven – (judged NSCLC in multidisciplinary tumour board meetings)	40
Size (cm)	2.2 (0.7–5.8)
Location	
Upper lobe	102 (56%)
Middle lobe	11 (6%)
Lower lobe	60 (33%)
Patients w 1 lesion	157
Patients w 2 lesions	13
Follow-up period (mo)	Median: 29, range: 8–72
Total dose - Gy	
48/ 4 fractions	120/183
50/ 5 fractions	13/183
52/ 4 fractions	50/183
56/ 4 fractions	1/183

One hundred and thirty-one tumors were biopsy proven based on transbronchial or CT-guided biopsy. Twelve tumors underwent inconclusive biopsy, and the remaining 40 tumors did not undergo biopsy and were judged to be non-small cell lung cancer by consensus in multidisciplinary tumor board meetings based on serial CT and PET-CT findings. The median follow-up period after SBRT was 29 months (range: 8–72).

### SBRT method

The SBRT technique used at our institution has been previously described [14, 15]. Patients were immobilized using one of 2 techniques: the Elekta BlueBAG vacuum cushion (Elekta AB, Stockholm, Sweden) with an abdominal compression plate, or the full Elekta BodyFIX system. A four dimensional (4D)-CT was acquired with phase-binning reconstruction software. The gross tumor volume (GTV) was delineated by the radiation oncologist on the 0% (peak inspiratory), 50% (peak expiratory), and maximum intensity projection (MIP) image sets, and their combined volume was used to generate the internal target volume (ITV). There was no expansion for microscopic disease. A 5-mm isotropic margin was added to form the planning target volume (PTV). The radiotherapy plan was calculated on the CT average image set and optimized using 7–10 beam angles. Intensity-modulated radiation therapy (IMRT) was used since 2009. The institutional policy was to deliver 48–52 Gy/4 fractions (fx) for peripheral NSCLC tumors (48 Gy if ≤3 cm, 52 Gy if > 3 cm) and 50 Gy/5fx for all central tumors (defined as tumors immediately adjacent to the esophagus, trachea, main stem bronchi, great vessels, and/or heart), regardless of size or histology. Plans were optimized to aim for ≥99% of the ITV to receive the prescription dose (ITV V100 ≥ 99%), and ≥ 99% of the PTV to receive 95% of the prescription dose (PTV V95 ≥ 99%). Radiotherapy plans were corrected for tissue inhomogeneity using the collapsed cone convolution algorithm.

Treatment was delivered using the Elekta Synergy units (Elekta AB, Stockholm, Sweden) equipped with the Elekta Synergy Beam Modulator (high resolution 4 mm multi-leaf collimator), a kilovoltage cone-beam CT (CBCT) image-guidance system and the Hexapod robotic couch permitting 6 degrees of freedom patient positioning.

### Chest CT examination

Patients were scheduled for chest CT follow-up in intervals of 3–4 months after SBRT for the first 3 years and every 6 months thereafter.

Chest CT studies were conducted using GE LightSpeed Plus or GE Lightspeed VCT 64 MDCT. The parameters used were the following: 120 kVp with tube current adjusted automatically, the beam pitch was 0.984:1, reconstruction thickness 0.625 mm, reconstruction interval 2.5 mm, scan field of view (FOV) 50 cm and display FOV adjusted to patient size, matrix 512 × 512 (pixel spacing: 0.933).

### Chest CT evaluation and CT patterns

Chest CTs were analyzed by a cardiothoracic radiologist with 15 years’ experience in thoracic imaging, a second-year cardiothoracic imaging fellow and a 4th-year medical student in consensus. We sought to classify the lung tumors on pre-treatment types and post-treatment CT patterns according to the *Fleischner Society* glossary of terms for thoracic imaging [16] and relevant literature on post radiation pulmonary CT patterns [6, 8, 17].

#### Pre-SBRT morphologic subtypes of lung tumor

1) solid nodule: rounded or irregular opacity with homogeneous soft tissue attenuation, well or poorly defined, measuring up to 3 cm in diameter (mass, if > 3 cm), 2) cavitary nodule: a nodule or mass with a gas-filled space seen as lucency or low attenuation area, measuring up to 3 cm in diameter (mass, if > 3 cm), 3) pure GGO nodule: rounded or irregular lesion with hazy increased attenuation in the lung that does not obliterate the bronchial and vascular margins, measuring up to 3 cm in diameter (mass, if > 3 cm), 4) mixed solid/GGO nodule: rounded or irregular lesion which consists of both ground glass and solid soft tissue attenuation components, measuring up to 3 cm in diameter (mass if > 3 cm).

### Post-SBRT CT patterns of lung injury

1) diffuse consolidation: homogeneous increase in pulmonary parenchymal attenuation that obscures the margins of vessels and airway walls occasionally with air-bronchogram, with no intervening areas of spared parenchyma, 2) peribronchial/patchy consolidation: patchy areas of consolidation located around the bronchovascular bundles with intervening areas of spared parenchyma, 3) consolidation and ground glass opacities (GGO): combination of areas with increased parenchymal attenuation that obscures the margins of vessels and airway walls and that does not obliterate the bronchial and vascular margins, 4) diffuse GGO: increased parenchymal attenuation that does not obliterate the bronchial and vascular margins with no intervening areas of spared parenchyma, 5) patchy GGO: increased parenchymal attenuation that does not obliterate the bronchial and vascular margins with intervening areas of spared parenchyma, 6) modified conventional pattern: consolidation associated with volume loss, traction bronchiectasis and architectural distortion, similar to, but less extensive than conventional radiation fibrosis, 7) nodule/mass-like pattern: focal consolidation limited around the treated tumor with a well-defined or irregular rounded morphology smaller than 3 cm (nodule) or larger than 3 cm (mass), 8) scar-like pattern: linear or band-like opacity in the region of the treated tumor associated with loss of volume, 9) “orbit-like pattern”: central solid nodule or mass surrounded by relatively normal lung parenchyma (skipped area) and more distally there is a peripheral circumferential band of consolidation, mimicking the appearance of the gravitational trajectory of an object around a central planet [18].

### Statistical analysis

Descriptive statistics frequency and percentages for categorical variables, median and range for continuous variables were used to describe the sample. The frequencies and percentages of pre-treatment lesions and post-treatment CT patterns were calculated. The time points of start and end of increase or decrease in size of the primary targeted lesions post SBRT were recorded. Similarly, the time points of start and end of increase or decrease of the overall volume of radiation injury - when this was inseparable from the targeted lesion - were recorded. The time points of start of each pattern post SBRT and duration of each pattern were also recorded. The period until stabilization without evidence of local or distant recurrence was calculated for each lesion. Stabilization of a CT pattern was defined as absence of change in morphology and size of radiation injury pattern over at least two consecutive follow-up CTs (8 months) until the end of the follow up period of the study. The time point that radiation-related rib fractures, local recurrence, distant or regional metastatic disease occurred post SBRT was also recorded. The statistical software SAS 9.4 was used for data manipulations and analyses.

## Results

Of the 183 treated tumors, “solid nodule” was the most common lesion pre-SBRT seen in 112 tumors (61%), followed by “solid mass” seen in 30 tumors (16%) and “part solid nodule” seen in 21 tumors (11%) (Table 2).

**Table 2:** Frequency of CT patterns of lung cancer before SBRT treatment

CT pattern	n/183	Frequency (%)
Cavitary nodule	4	2
Mixed solid /GGO mass	4	2
Cavitary mass	5	3
Pure GGO nodule	7	4
Mixed solid /GGO nodule	21	11
Solid mass	30	16
Solid nodule	112	61

**Fig. 2:**
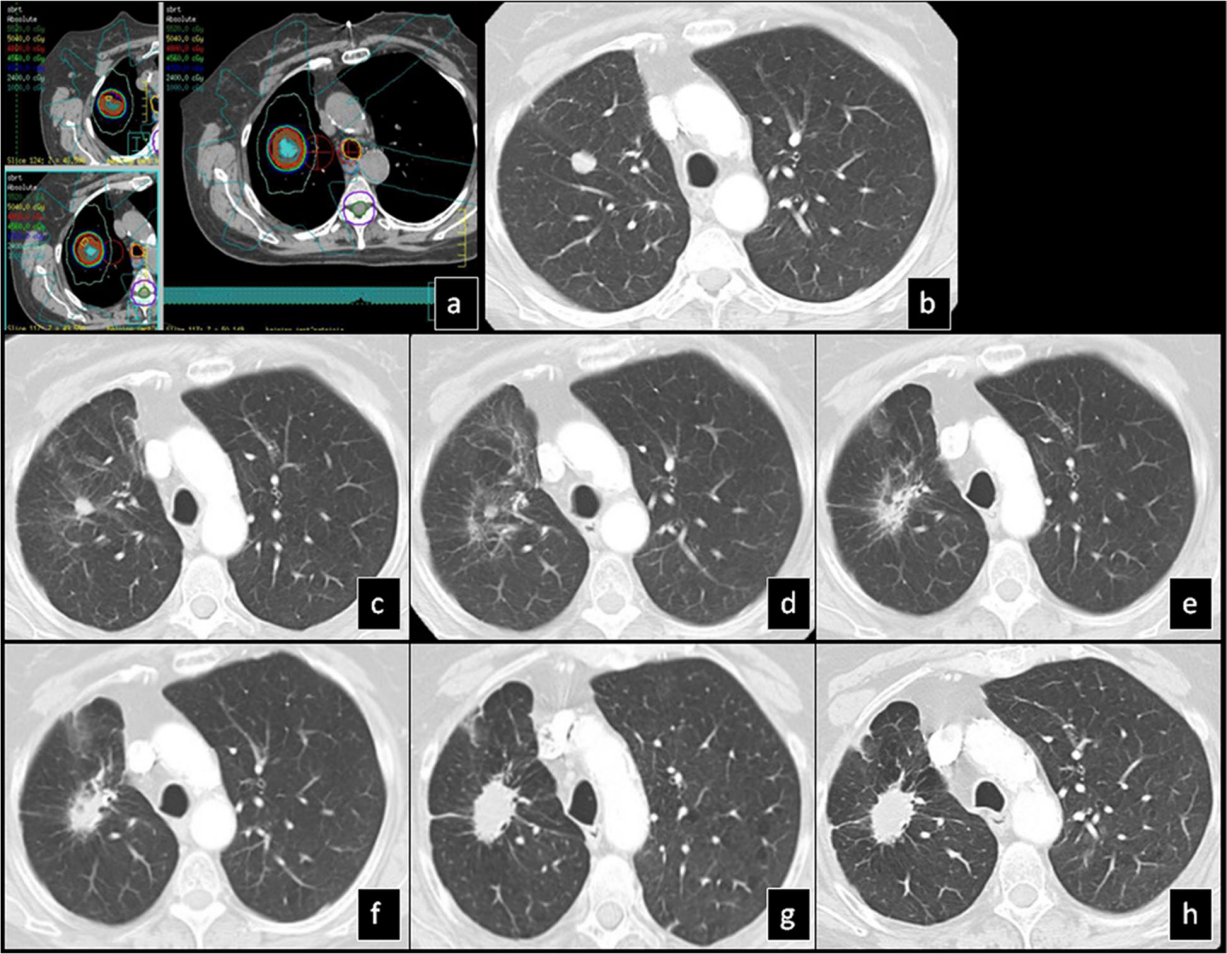
SBRT-treated lung cancer demonstrates stabilization at 61 months: **a**) Isodose multiplanar images for SBRT planning. **b**) Pre-SBRT CT shows a solid nodule. **c**) 10 months (mo) post-SBRT the primary lesion slightly decreased in size with development of diffuse ground glass opacity surrounding the nodule. **d**) 24 mo post-SBRT “the orbit sign” pattern developed. 40 (**e**) and 49 mo (**f**) post-SBRT there was gradual increase in density “filling in” the area between the irradiated nodule and the surrounding curvilinear density gradually obscuring the “orbit sign”. **g**) 61 mo post-SBRT there was stabilization of “mass-like” pattern. h) 72 mo post-SBRT there was no interval change

During follow-up of the lung tumors, 119 of the tumors decreased in size starting at 4 months. Eleven out of the 183 tumors increased in size starting at 8 months and continued to increase up to 13 months (Table 3). In 90 lesions that were inseparable from the surrounding post radiation changes, the overall volume of radiation injury increased starting at 9 months and continued to increase up to 13 months (Fig. 2). In 115 lesions that were inseparable from the surrounding post radiation changes, the overall volume of radiation change decreased starting at 9 months and continued to decrease up to 14 months post-SBRT (Table 3).

**Table 3:** Time points post-SBRT of increase or decrease of primary lesion or overall surrounding consolidation

	Tumors (n)	Starting time point of post-SBRT changes in months, median (range)	Ending time point of post-SBRT changes in months, median (range)
Primary lesion increases	11	8 (4–21)	13 (4–21)
Primary lesion decreases	119	4 (4–17)	4 (4–27)
Overall surrounding consolidation increases	90	9 (4–55)	13 (4–60)
Overall surrounding consolidation decreases	115	9 (4–70)	14 (4–70)

The frequency of the different CT patterns of post-SBRT radiation lung injury is illustrated in Table 4.

**Table 4:** Frequency of CT patterns of lung cancer post-SBRT treatment

CT pattern	n/183	Frequency (%)
GGO diffuse	6	3
Scar-like pattern	28	15
Consolidation diffuse	29	16
Mass-like pattern	34	19
Orbit sign pattern	39	21
GGO patchy	44	24
Modified conventional pattern	71	39
Consolidation + GGO	75	41
Consolidation peribronchial/patchy	77	42
Nodule-like pattern	126	69

Many tumors demonstrated more than one or multiple CT patterns of post-SBRT radiation change

**Fig. 3:**
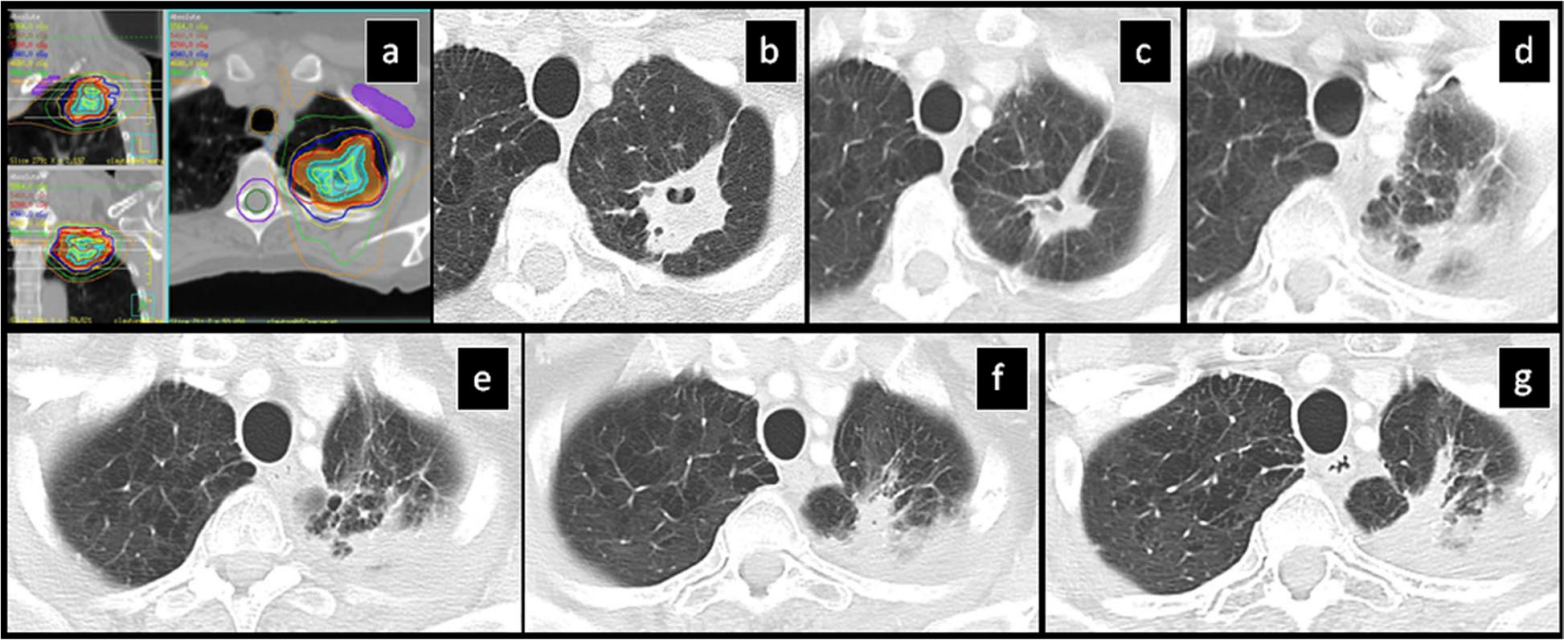
SBRT-treated lung cancer demonstrates local recurrence at 35 months: **a**) Isodose multiplanar images for planning. **b**) Pre-SBRT CT shows a cavitary mass in the left upper lobe. **c**) 6 mo post-SBRT, the lesion decreased in size. **d**) 16 mo post-SBRT, the primary lesion is obscured by surrounding consolidation and mild GGO. **e**) 20 mo post-SBRT there is architectural distortion in keeping with “modified conventional pattern”. **f**) 30 and g) 36 mo post-SBRT, the radiation changes became denser and more well-defined retaining the “modified conventional pattern”

**Fig. 4:**
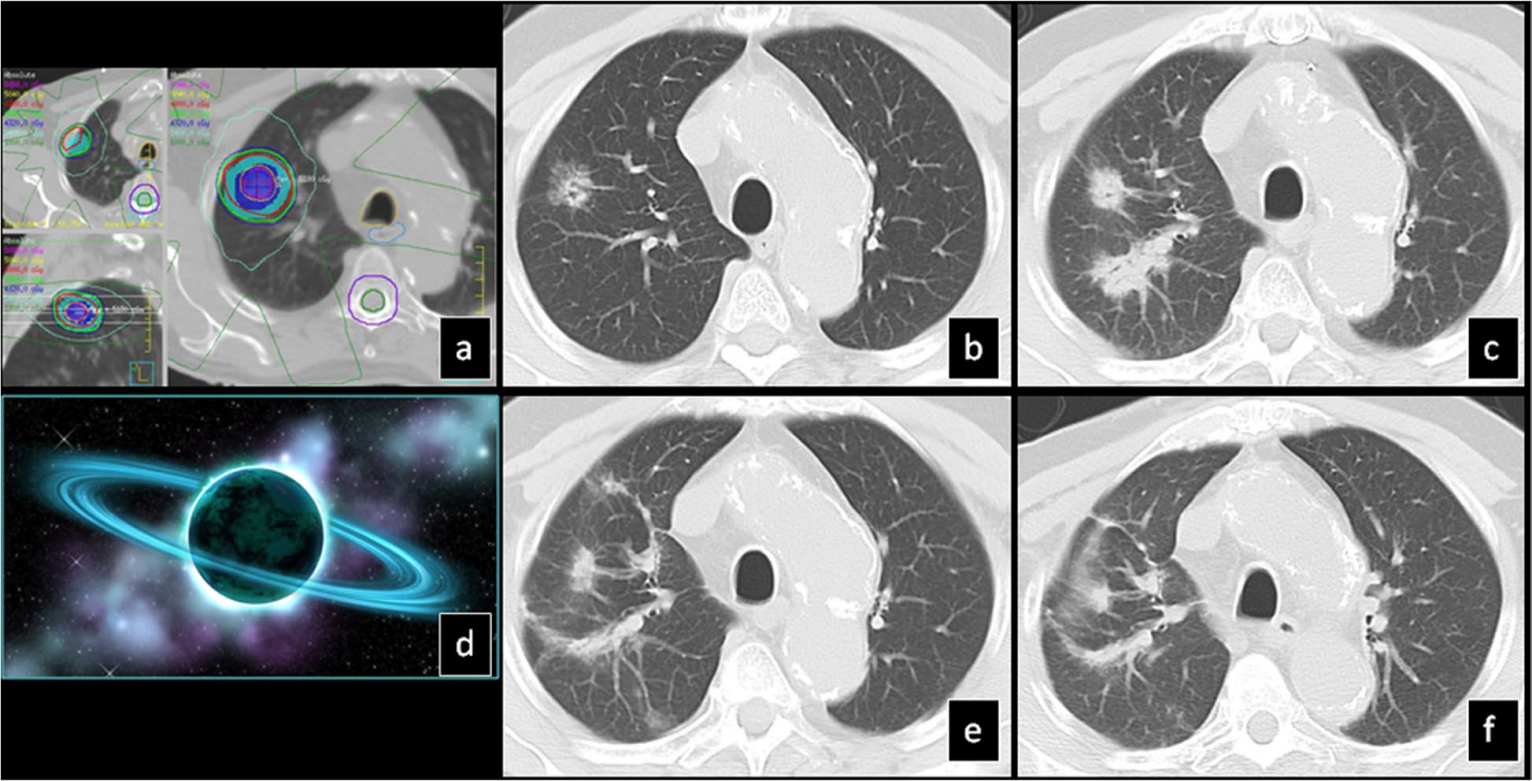
SBRT-treated lung cancer continues to decrease in size at 19 months: **a**) Isodose multiplanar images for planning. **b**) Pre-SBRT CT shows a part-solid nodule. **c**) 6 mo post-SBRT the size is stable, however the nodule is denser and there is surrounding focal peribronchial consolidation away from the nodule. **d**, **e**) 10 mo post-SBRT there is decrease in size of the primary lesion and development of homogeneously thick curvilinear density at a radius away from the center of the nodule in keeping with the “orbit-sign” (**d**). **f**) 19 mo post-SBRT there is further decrease in size of the primary lesion and mild shrinkage of the surrounding “orbit-sign”

**Fig. 5:**
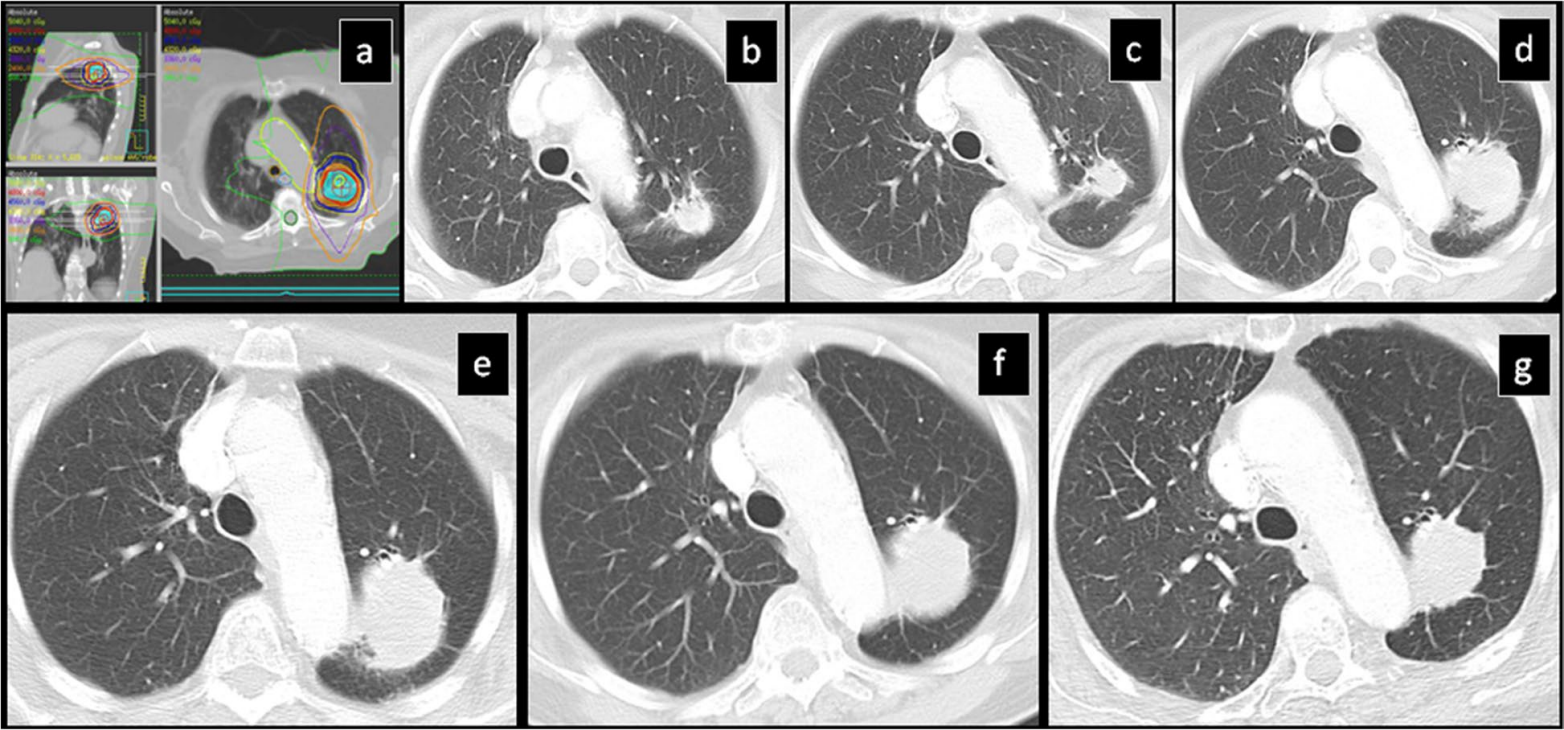
SBRT-treated lung cancer continues to decrease in size at 49 months: **a**) Isodose multiplanar images for planning. **b**) pre-SBRT CT shows a solid nodule. **c**) 6 mo post-SBRT, the primary lesion slightly decreases in size and there is mild adjacent peribronchial consolidation. d) 9 mo post-SBRT, the lesion significantly increases in overall size with mild surrounding peribronchial consolidation. **e**) 18 mo post-SBRT, the lesion size is unchanged and there is improvement of the surrounding peribronchial consolidation, **f**) 22 mo post-SBRT, there is mild decrease of the lesion size and resolution of surrounding peribronchial consolidation, **g**) 49 mo post-SBRT, there is further mild decrease in lesion size

There was a significant overlap of CT patterns affecting the same tumor during multiple follow up CTs. The most frequent CT pattern post-SBRT was the “nodule-like pattern” (69%), followed by the “peribronchial/patchy consolidation” (42%), the “consolidation with GGO” (41%) and the “modified conventional pattern” (39%) (Fig. 3). “Patchy GGO”, the “orbit sign pattern” (Fig. 4) and “mass-like pattern” (Fig. 5) were seen in 24, 21 and 19% of the tumors respectively.

Time points of appearance of CT patterns post-SBRT and total duration are presented in detail in Table 5.

**Table 5:** Time points of appearance of CT patterns post-SBRT and total duration (months)

	Starting time point of post-SBRT changes in months, median	Duration of post-SBRT changes in months, median (range)
Consolidation peribronchial / patchy	4	4 (3–31)
GGO patchy	4	5 (3–31)
Nodule-like pattern	4	7 (3–34)
Consolidation diffuse	5	4 (3–25)
Consolidation + GGO	5	6 (3–36)
GGO diffuse	6	4 (3–20)
Orbit sign pattern	8	9 (3–32)
Mass-like pattern	8	15 (3–59)
Modified conventional pattern	12	24 (3–57)
Scar-like pattern	12	29 (3–59)

**Fig. 6:**
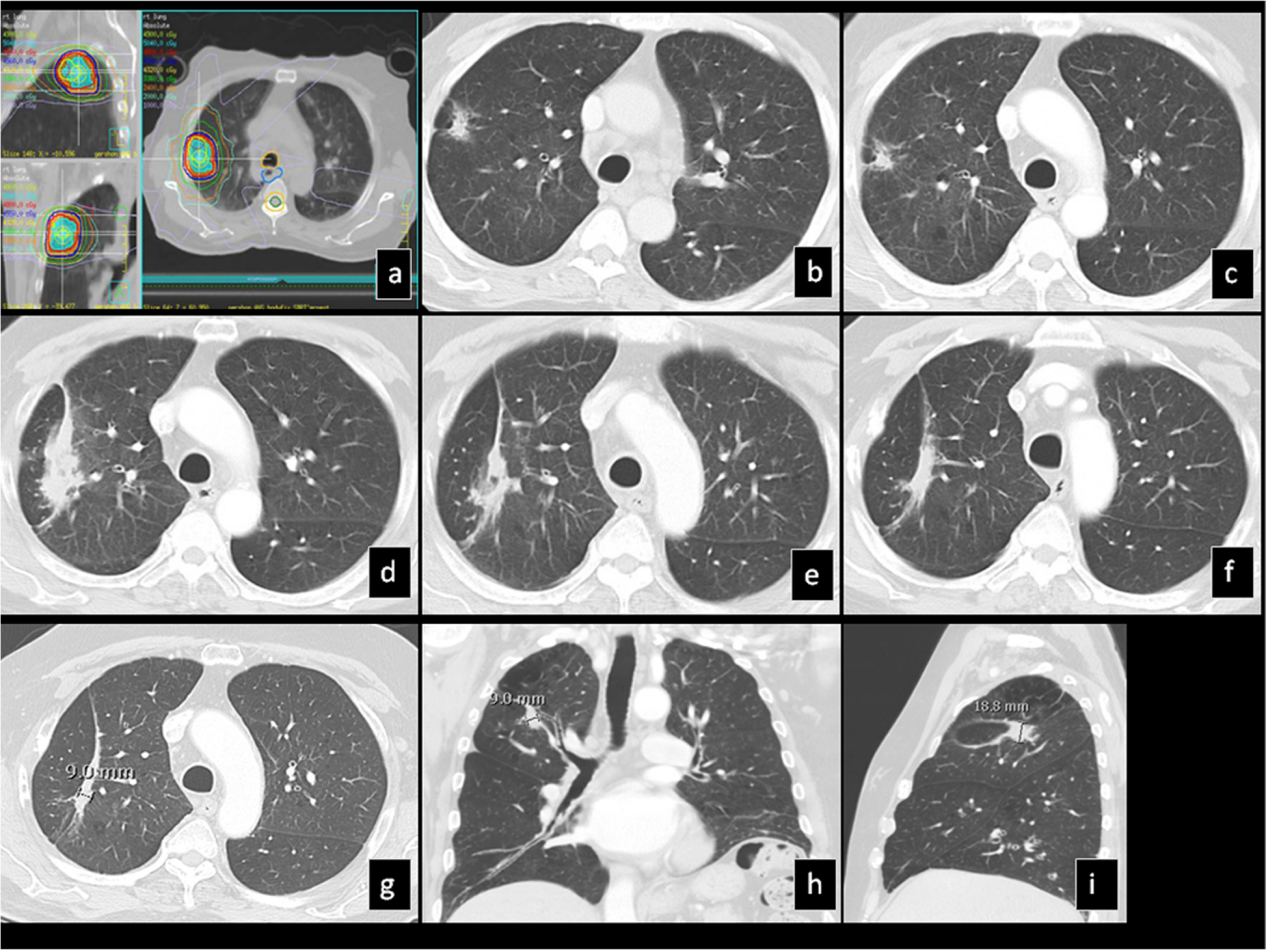
SBRT-treated lung cancer demonstrates stabilization at 35 months: **a**) Isodose multiplanar images for planning. **b**) Pre-SBRT CT shows a part-solid nodule. **c**) The nodule slightly decreased in size 9 months post-SBRT. **d**) 13 mo post-SBRT, the overall volume of the surrounding radiation changes obscuring the nodule increased in size with a pattern resembling “peribronchial consolidation”. **e**) 23 mo post-SBRT, the radiation changes decreased in size demonstrating a “scar-like” pattern and **f**) in 35 mo, there was stabilization of the “scar-like” pattern. **g**) 48 mo post-SBRT there was no change. Coronal (**h**) and sagittal (**i**) reconstruction at 48 mo (same time point as in fig. 5f) shows “scar-like pattern” after stabilization. The “width” of the lesion is small in 2 of the 3 planes (axial (**g**) and coronal (**h**)) compared to the third plane (sagittal (**i**)). To correctly identify the “scar-like pattern” careful evaluation of all 3 planes is needed as the lesion usually looks “bulkier” in one plane giving the false impression of a more “mass-like” pattern

Time to stabilization in 91 lesions was 20 months ranging from 8 to 62 months. The most common pattern at stabilization of findings was the “modified conventional pattern” (49%), followed by the “scar-like pattern” (23%) (Fig. 6) and the “mass-like pattern” (12%) (Table 6).

**Table 6:** Frequency of CT patterns of lung cancer post-SBRT treatment at stabilization

CT pattern	n/90	Frequency (%)
Consolidation peribronchial/patchy	1	1.1
Consolidation + GGO	1	1.1
Consolidation diffuse	3	3.3
Orbit sign pattern	4	4.4
Nodule-like pattern	5	5.5
Mass-like pattern	11	12.2
Scar-like pattern	21	23.3
Modified conventional pattern	44	48.8

**Fig. 7:**
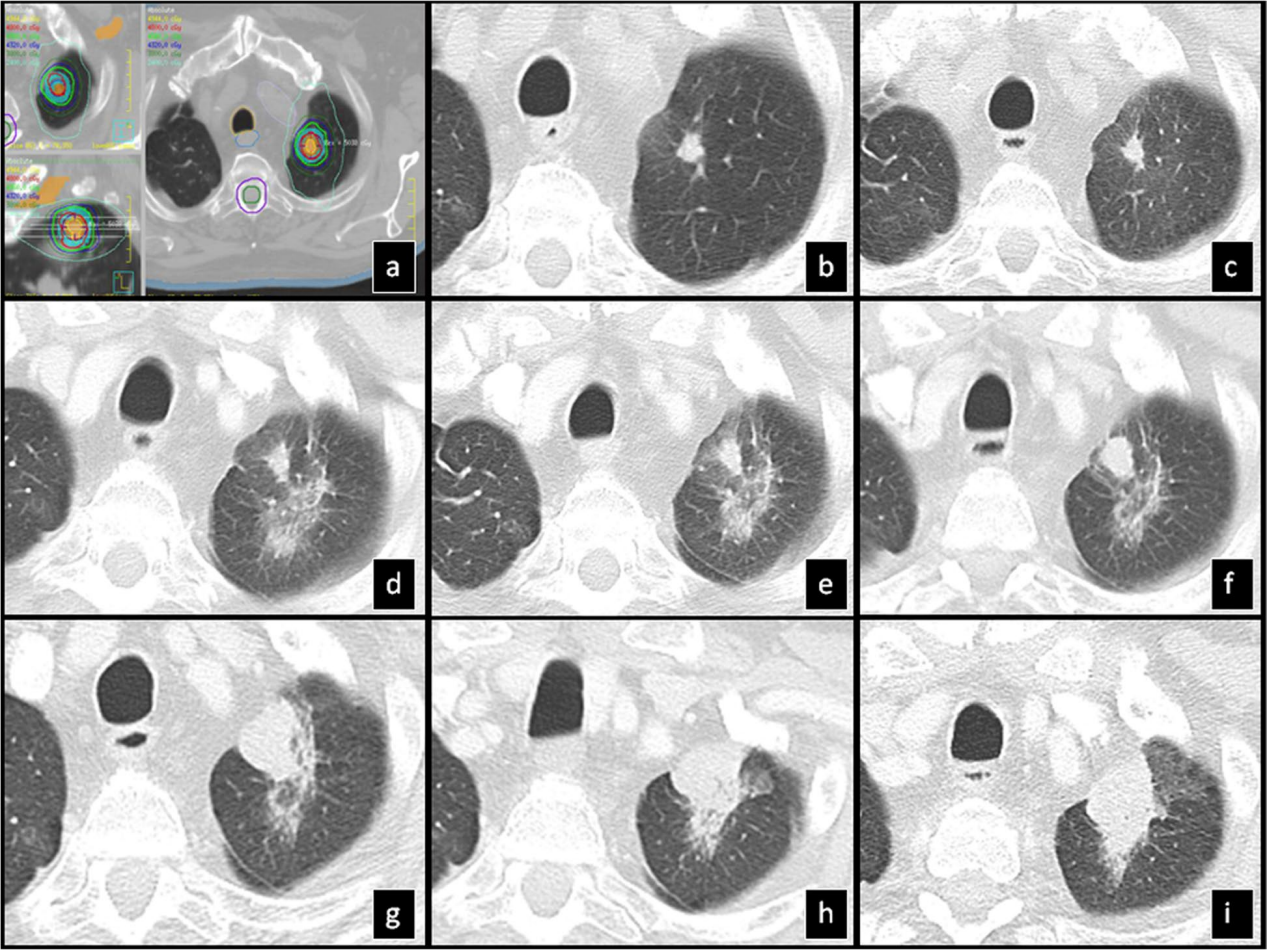
SBRT-treated lung cancer demonstrates local recurrence at 35 months: **a**) Isodose multiplanar images for planning. **b**) pre-SBRT CT shows a solid nodule. **c**) 8 mo post-SBRT, the nodule has not changed significantly in size, however there is new surrounding GGO. **d**) 13 mo post-SBRT the lesion is stable and there is surrounding patchy GGO and peribronchial consolidation. **e**) 17 mo post-SBRT, there is slight increase of the nodule size with stable patchy GGO and consolidation. **f**) 26 mo post-SBRT, there is further mild interval increase of the nodule size and slight improvement of the surrounding GGO and consolidation. **g**) 31 mo post-SBRT, there is further interval increase of the lesion size (now a mass) and increased density of the adjacent radiation changes. **h**) 35 mo post-SBRT there is further increase of the primary lesion partially obscured by the surrounding radiation changes which demonstrate increased density. Local recurrence was established at this point. **i**) 39 mo post-SBRT, there is further increase of the primary lesion which is now inseparable for surrounding radiation changes which demonstrate further increased density

Local recurrence occurred in 15 lesions at a median period of 18 months (7–52). The most common pattern at the time of diagnosis of local recurrence was the “mass-like pattern” (7 cases) (Fig. 7), followed by the “nodular pattern” (5 cases), “modified conventional pattern” (2 cases) and “diffuse consolidation” in 1 case. Distant metastasis occurred in 34 cases at a median period of 18 months (range: 3–66) and radiation-related insufficiency rib fractures occurred in 78 cases at a median period of 19 months (range: 9–68).

## Discussion

Understanding the natural history of evolution of CT changes post SBRT helps differentiating fibrosis from local recurrence or other complications such as pulmonary infection or even development of a new metachronous cancer [19].

We found the median time interval to the earliest CT changes to be 4 months post SBRT, which is longer than the reported 4 weeks after completion of conventional radiotherapy [20]*.* This may be secondary to the higher dose used in SBRT since it has been reported that as the dose per fraction increases, the higher the probability of late phase injury [6]. However since patients had their first follow up chest CT at 3 or 4 months post SBRT it would have been impossible to document any earlier changes as an earlier than 3 months chest CT would have exposed patients to unnecessary radiation burden. The earliest CT patterns detected were peribronchial patchy consolidation, patchy GGO and nodule-like pattern at 4 months. Diffuse consolidation and combination of consolidation and GGO were seen at 5 months, diffuse GGO started at 6 months and mass-like pattern and orbit sign were first seen at 8 months. The latest CT patterns were the scar-like and modified conventional patterns, which were seen at 12 months. This timeline agrees with earlier studies that have classified changes in “early” (before 6 months) and “late” changes (after 6 months) [20–22]. Early changes according to Ikezoe et al. [17] include a) the diffuse consolidation pattern, b) the diffuse GGO pattern, c) the patchy GGO pattern, d) the patchy consolidation and GGO pattern and e) no change. All these changes were found to start at 4–5 months post-SBRT in our study. According to Dahele et al., the median time interval to the initial CT changes post-SBRT is 17 weeks (approximately 4 months) like in our study [21]. The late changes have been classically classified according to Koenig et al. [23] including a) the modified conventional pattern, b) the scar-like pattern, c) the mass-like pattern and d) no change. The 3 first patterns were identified in our study at 8 and 12 months in agreement with the literature.

Two other patterns observed in our study that have not been used in previous classifications are the “nodule-like” pattern and the “orbit sign”. The nodule-like pattern was described when the lesion retained a nodular morphology post-SBRT. The lesion usually presented as a solid nodule even if the initial lesion was GGO or subsolid in density. This CT pattern was the most frequent post SBRT and was seen as early as 4 months post SBRT. The other novel sign in our study was the “orbit-sign” which was initially seen at 8 months post SBRT and presented as a central solid nodule or mass surrounded by relatively normal lung parenchyma (skipped area) and more distally demonstrating a peripheral circumferential band of consolidation remaining at the margin of the targeted volume, however at a short distance from the isocenter and typically conforming to the shape of the tumor [18, 24]. Similar patterns have been described previously as “ground glass opacity surrounding the irradiated lesion and failing to fill in all the targeted volume” [6*,* 24]. This may be attributed to the fact that in SBRT, a steeper gradient is generated between the periphery of the planned target volume (high-dose area) and normal adjacent structures (low dose areas) as the targeted tumors are usually small in size and the beam focus is therefore thinner [25]. Moreover, SBRT delivers the focused dose to the tumor via multiple beams, resulting in atypical patterns of lung injury that may be seen away from the original site and can be mistaken for other disease entities [26].

A major diagnostic dilemma in interpreting post-SBRT changes is the expected period that either the treated lesion or the combination of the treated lesion and the adjacent inseparable radiation changes may continue to grow post treatment without this representing local recurrence. In this study, we calculated the period that each treated tumor or the tumor and the inseparable surrounding post-radiation changes started and stopped changing in size. Our results showed that the combination of irradiated tumor with the inseparable adjacent post radiation changes may start decreasing in size at a median time interval of 9 months and may continue to decrease in size up to 70 months post SBRT. Interestingly although the inseparable tumor and surrounding post radiation changes may stop increasing at a median interval time of 14 months, rarely they might continue to increase up to 60 months (5 years) post treatment without this representing local recurrence. Dahele et al. also reported that irradiated lesions may continue to evolve for more than 2 years after SBRT although evolution of up to 4 years was rarely seen [21]. On the other hand Linda et al., reported that the size of SBRT-induced consolidation does not increase beyond 12 months unless it harbors local recurrence [6]. The findings in our study support the presence of a prolonged type of lung injury manifested initially as inflammation and subsequently replaced by fibrosis, which may depend on the absorbed radiation dose, the number of fractions into which the absorbed dose is divided and the size of the individual dose per fraction [27, 28].

The median interval time to stabilization was 20 months ranging from 8 to 62 months. The most common patterns were the “modified conventional pattern” followed by the “scar-like pattern” and the “mass-like pattern”. Similarly Trovo et al. reported that from 13 to 18 months post SBRT they found 44% conventional pattern and 28% mass-like pattern [20]. Dahele et al. reported that beyond 6 months post SBRT they found 71% conventional pattern, scar-like pattern in 11% and only 7% mass-like fibrosis [20]. The “scar-like” CT pattern was described in 15% of cases in another study [29]. The “scar-like” pattern may be appreciated in 1 or 2 reconstruction CT planes, while in the third plane (axial, coronal or sagittal) it may look flat, occupying a large area. Therefore, careful assessment of all 3 planes is recommended in accurately identifying this pattern (Fig. 6). Mass-like pattern can be challenging as it may mimic local recurrence. It represents a well-defined mass-like consolidation which can be larger than the original tumor, does not have air-bronchograms and lacks straight margins [19]. This is attributed to the fact that SBRT is delivered in a 3D spherical volume with a gradient between the high dose at the periphery of the target volume and the low dose within normal adjacent tissue resulting in the shape of the SBRT-induced injury conforming more tightly to the shape of the tumor. This contrasts with conventional radiotherapy where radiation injuries show a linear shape and distinct margins on CT that correspond to the simple shape of the dose distribution of irradiated lungs and the distinct boundary between the non-irradiated and irradiated lung, respectively [6]. We also observed that the orbit sign would evolve with time to mass-like fibrosis pattern as there was gradual filling in of the adjacent - “initially spared” - lung parenchyma with increased density (Fig. 2).

**Fig. 8:**
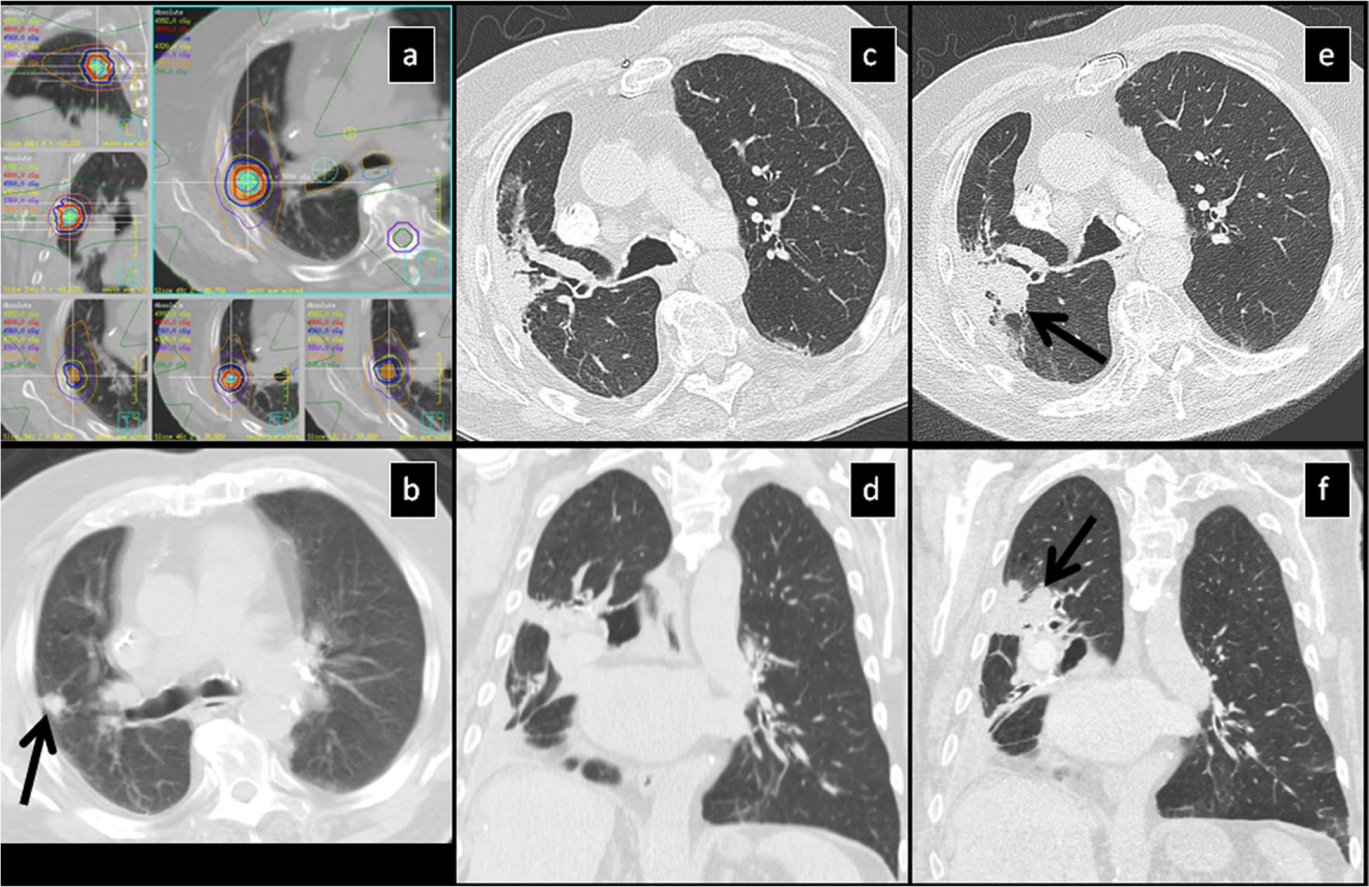
SBRT-treated lung cancer demonstrates local recurrence at 5 years: **a**) Isodose multiplanar images for planning. **b**) pre-SBRT, CT shows a solid nodule (black arrow). Axial (**c**) and coronal plane (**d**) chest CT 3 years post-SBRT show “modified conventional pattern” that has stabilized. 5 years post-SBRT axial (**e**) and coronal (**f**) images show increased soft tissue density bulging on one side of the lesion (posteriorly on axial plane and superiorly on coronal plane, black arrows) in keeping with local recurrence

Local recurrence occurred in 15 lesions at a median period of 18 months post-SBRT and in all cases, there was rapid progressive enlargement in 2–3 consecutive CTs within a period of 6–12 months (Fig. 7). The most common pattern was the mass-like pattern, followed by the nodular pattern. In the 2 cases where modified conventional pattern was still seen at the time that local recurrence was diagnosed, there was central “filling” of the post radiation fibrotic changes with homogeneous soft tissue density without air-bronchogram or nodular bulging on one side of the lesion (Fig. 8). Therefore, the rapid evolution to nodule or mass-like pattern without internal air-bronchogram and accompanied by increase in overall volume of the lesion is highly suspicious of local recurrence. The following 6 features representing local recurrence have been reported previously: a) an enlarging opacity at the site of the treated tumor, b) a sequentially enlarging opacity, c) loss of linear margin, d) development of a bulging margin of the post radiation changes, e) effacement of previously noted air-bronchogram and f) enlarging opacity without air-bronchogram, particularly after 12 months [19, 30].

Our study has inherent limitations associated with its retrospective nature and lack of evaluation of interobserver agreement. Furthermore, as the first follow up chest CT was obtained at 3–4 months post initiation of SBRT, we may have missed the exact time point where any post SBRT changes may have truly started. However, it would be impossible to capture any post SBRT changes earlier as this would have exposed patients to a significant radiation burden. Finally, there was absence of biopsy confirmation in 40 out of 183 tumors.

In conclusion, post-SBRT lung injury may present with different CT patterns which may overlap in a single patient and usually have specific timelines of presentation and duration. Commonly one CT pattern will evolve to another CT pattern with time and SBRT-treated tumors may initially increase in size until they stabilize or eventually shrink in size. Post- SBRT changes may evolve over a prolonged period until stabilization, and this may vary significantly and rarely can last more than 4 years. Familiarity of the thoracic radiologist with the specific CT patterns and timelines of presentation of post-SBRT changes may significantly increase the confidence towards an accurate diagnosis differentiating local recurrence from radiation changes. Moreover, it will help obviate or decrease the number of unnecessary invasive high-risk complication procedures in a population with increased comorbidities.

## Data Availability

The datasets used and/or analysed during the current study are available from the corresponding author on reasonable request.

## Abbreviations

SBRT: Stereotactic body radiotherapy
GGO: Ground glass opacity
CT: Computed tomography
NSCLC: Non-small cell lung caner
